# Whole genome sequencing for drug resistance determination in *Mycobacterium tuberculosis*

**DOI:** 10.4102/ajlm.v8i1.801

**Published:** 2019-02-21

**Authors:** Shaheed V. Omar, Lavania Joseph, Halima M. Said, Farzana Ismail, Nabila Ismail, Thabisile L. Gwala, Nazir A. Ismail

**Affiliations:** 1 Centre for Tuberculosis, World Health Organization TB Supranational Reference Laboratory Network, National Institute for Communicable Diseases, National Health Laboratory Service, Johannesburg, South Africa; 2 Department of Medical Microbiology, University of the Free State, Bloemfontein, South Africa; 3 Department of Medical Microbiology, University of Pretoria, Pretoria, South Africa

## Abstract

South Africa remains challenged with a high tuberculosis burden accompanied by an increase in drug resistant cases. We assessed the use of the Illumina MiSeq, a next-generation sequencing platform for whole genome sequencing, followed by bioinformatic analysis using a commercial software package to determine resistance to selected drugs used for *Mycobacterium tuberculosis* treatment in our setting. Whole genome sequencing shows potential as a diagnostic platform for the detection of drug resistance in *Mycobacterium tuberculosis* with the provision of information for several drugs simultaneously.

## Introduction

Drug-resistant tuberculosis poses a significant challenge to tuberculosis control programmes in high burden settings.^1^ Undiagnosed drug resistance leads to further transmission, poor patient outcomes and potential for amplification of drug resistance, impeding the World Health Organization’s (WHO) strategy to end tuberculosis by 2035. The drug-resistant tuberculosis outbreaks in Tugela Ferry^2^ and other regions of South Africa^3^ highlight the need for early and accurate diagnosis of drug resistance.

Often, comprehensive phenotypic baseline testing is not available nor is a robust surveillance programme in place to inform regimen changes appropriate to local resistance profiles.^4^ A paradigm shift is needed in the approach to diagnosis and surveillance of drug-resistant tuberculosis to ensure that new drug potential is not lost due to the evolution and spread of resistant strains. Molecular testing such as the line probe assay and Xpert MTB/RIF assay (Cepheid, Sunnyvale, California, United States) show potential superiority in overall performance over phenotypic drug susceptibility testing (DST).^5,6^ A targeted sequencing approach for resistance detection in *Mycobacterium tuberculosis* by application of next-generation sequencing benchtop platforms showed good performance in terms of sensitivity.^7^ With the decreasing cost of next-generation sequencing, whole genome sequencing (WGS) could be applied for this purpose as an alternative to conventional phenotypic methods.^8,9^ The direct benefit of WGS is its ability to provide organism identification, strain relatedness and a drug resistance profile for characterised resistance-conferring mutations. In addition, WGS may be useful for resistance determination for newer drugs lacking validated DST such as bedaquiline and delamanid, utilising information available for the genetic basis associated with resistance *in vitro* to these novel drugs.^10,11^

We assessed the use of the Illumina MiSeq^®^ sequencing,^12^ followed by bioinformatic analysis using a commercial software (CLC Genomics Workbench, Qiagen, Venlo, The Netherlands) for drug resistance determination at the National Tuberculosis Reference Laboratory in South Africa.

## Methods

### Ethical considerations

Ethical approval was not required for this laboratory-based study as only anonymised isolates were used.

### Sample selection

Twenty geographically diverse clinically isolated *M. tuberculosis* strains, with varying resistance profiles and spoligotype patterns, isolated between June 2012 and January 2013 were selected for this pilot evaluation (Table 1). Laboratory processing for culture, smear microscopy and DST were performed according to WHO guidelines.^13^ Six of the 20 isolates had discordant phenotypic results between initial and repeat testing to either the fluoroquinolones or pyrazinamide.

**TABLE 1:** Summary of performance for drug resistance determination using the MGIT960, MTBDRplus assay and whole genome sequencing.

Isolate† phenotype	Isolates (*n* = 20)	Rifampicin			Isoniazid			Fluoroquinolone ofloxacin & moxifloxacin		Aminoglycosidekanamycin & amikacin		Pyrazinamide	
		*Phenotype*	MTBDR*plus*	*WGS*	*Phenotype*	MTBDR*plus*	*WGS*	*Phenotype*	*WGS*	*Phenotype*	*WGS*	*Phenotype*	*WGS*
Concordant Susceptibility	6	S	S	S	S	S	S	S	S	S	S	S	S
Discordant in only 1 test	1	S	S	S	S‡	*inhA*^*mut1*^	*InhA*^*C-15T*^	S	S	S	S	S	S
Multidrug resistant	5	R	*rpoB*^*Mut2a*^	*rpoB*^*H526Y*^	R	*katG*^*mut2*^*/inhA*^*mut1*^	*katG*^*S315T*^*/inhA*^*c-15t*^	S	S	S	S	R	*pncA*^*H71Y*^
		R	S§	*rpoB*^*L511P*^	R	*inhA*^*mut3a*^	*inhA*^*t-8c*^	S	S	S	S	S	S
		R	*rpoB*^*mut3*^	*rpoB*^*S531L*^	R	*inhA*^*mut1*^	*inhA*^*c-15t*^	S	S	S	S	S	S
		R	*rpoB*^*mut2a*^	*rpoB*^*H526Y*^	R	*inhA*^*mut1*^	*inhA*^*c-15t*^	S	S	S	S	S	S
		R	*rpoB*^*mut3*^	*rpoB*^*S531L*^	R	*inhA*^*mut1*^	*inhA*^*c-15t*^	S	S	S	S	S	*pncA*^*T114M*^
Pre-extensively drug resistant	1	R	*rpoB*^*mut3*^	*rpoB*^*S531L*^	R	*katG*^*mut2*^	*katG*^*S315T*^	R	*gyrA*^*D94G*^	S	S	R	*pncA*^*C14R*^
Extensively drug resistant	1	R	*rpoB*^*WT2missing*^	*rpoB*^*H526L*^	R	*katG*^*mut3*^	*katG*^*S315T*^	R	*gyrA*^*A90V*^	R	*rrs*^*a1401g*^	R	*pncA*^*del373-387*^
Discordant Pyrazinamide	3	S	S	S	S	S	S	S	S	S	S	S‡	S
		S	S	S	S	S	S	S	S	S	S	S‡	S
		S	S	S	S	S	S	S	S	S	S	S‡	S
Discordant Fluoroquinolone	3	S	S	S	S	S	S	S‡	S	S	S	S	S
		S	S	S	S	S	S	ofloxacinR‡	S	S	S	S	S
		S	S	S	S	S	S	ofloxacinR‡	S	S	S	S	S

S, susceptible; R, resistant; MGIT, Mycobacterial Growth Indicator Tube; WGS, whole genome sequencing.

lower case represents nucleotide position change and uppercase amino acid position change (single abbreviation).

† , Spoligotype profiles included – Beijing, Latin-American-Mediterranean (LAM), Central Asian Strain (CAS), East African-Indian (EAI) and East African-Indian – Somalian (EAI1).

‡ , Discordant result between initial and repeat testing.

§ , Incorrectly classified as susceptible by MTBDR*plus* assay.

### Routine laboratory phenotypic testing

Phenotypic DST was performed on the BACTEC Mycobacterial Growth Indicator Tube (MGIT) 960 system (Becton Dickinson Diagnostic Systems, Sparks, Maryland, United States) following the manufacturer’s recommendation. First and second-line anti-mycobacterial drugs (rifampicin, isoniazid, ofloxacin, moxifloxacin, ptyrazinamide, amikacin, and kanamycin) were tested following the WHO 2012 Policy Guidelines.^14^ Replicate testing was performed on any isolate resistant to pyrazinamide or second-line drugs on initial testing.

### Next-generation sequencing

**FIGURE 1:**
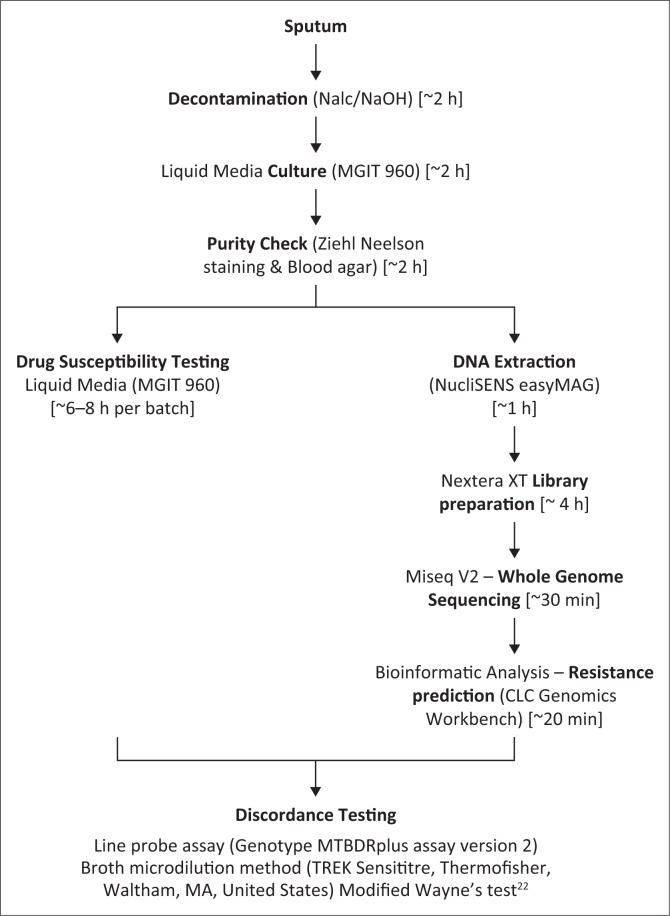
Operational workflow for phenotypic drug resistance determination versus resistance prediction by whole genome sequencing. The batch size is calculated at 20 isolates [estimated hands-on time].

WGS was performed using the MiSeq version 2 kit (Illumina, San Diego, California, United States). In brief, DNA was extracted using the NucliSENS easyMAG system (BioMérieux, Marcy-l’Étoile, France) from a 200 *µ*l aliquot of heat- inactivated, MGIT-cultured isolate and concentrations quantified using the Qubit dsDNA HS (high sensitivity) assay (Life Technologies, Carlsbad, California, United States). Libraries were prepared using Nextera XT kit (Illumina, San Diego, California, United States) following the manufacturers’ protocol with one modification (Figure 1). The modification deviated at the normalisation step, where the indexed DNA libraries concentrations were quantified as described above and normalised to 4 nM by addition of Tris-Cl (10 nM, pH8.5 with 0.1% Tween20). Thereafter, the indexed libraries of all 20 isolates were pooled to a final concentration of 12 pM and loaded onto the MiSeq for sequencing.

### Bioinformatic analysis of WGS data

CLC Genomics Workbench version 6.0.1 (Qiagen, Venlo, the Netherlands) was used for bioinformatic analysis. Variant tables for genetic targets associated with resistance to rifampicin, isoniazid, fluoroquinolones (ofloxacin and moxifloxacin), pyrazinamide, aminoglycosides (amikacin and kanamycin), bedaquiline and delamanid (Table 2) were generated using the *Map Reads to Reference* tool and *Quality-based Variant Detection* algorithm on CLC Genomics Workbench using the H37Rv Sanger reference genome (GenBank NC000962.3). The following cut-offs were applied to call a single nucleotide polymorphism or insertion/deletion: a minimum paired coverage depth of five times (5×), frequency of > 70% and a Phil’s Read Editor, or PHRED, quality score of ≥ Q20 (≥ 99% accuracy) at the variant position and neighbouring nucleotides within a radius of five base pairs. To ensure that an isolate was truly wild-type for a specific gene target, we further ran the *Create Statistics for Target Regions* on CLC Genomics Workbench to ensure that the entire length of the gene investigated was completely sequenced. Since no thresholds have been formally established for bioinformatic analysis, we utilised less stringent parameters than those previously described.^15^

**TABLE 2:** Table detailing first-line, second-line and novel tuberculosis drugs, their resistance-associated genes and their length.

Drug	Resistance associated Gene	Length
Rifampicin	*rpoB*	3519 bp
Isoniazid	*katG* *inhA* promoter (fabG1/mabA)	2223 bp 140 bp†
Ofloxacin/Moxifloxacin	gyrA *gyrB*	2517 bp 2028 bp
Amikacin/Kanamycin	*rrs* *eis* promoter	1537 bp 36 bp†
Pyrazinamide	*pncA* /(pncA promoter)	561 bp (100 bp)
Bedaquiline^11,23^	*atpE* *Rv0678*	246 bp 498 bp
Delamanid^19^	*ddn* *fgd1*	456 bp 1011 bp

† , The *inhA and eis* promoter regions were additionally annotated on the reference genome.

Association of mutations as resistance predictors were identified using the TB Drug Resistance Mutation Database (TBDReaMDB) database^16^ primarily. If a mutation was not listed, literature, including newer published databases such as TBProfiler and PhyResSE, was surveyed to identify the association.^17,18^ Putative mutations associated with the novel drugs bedaquiline and delamanid were exclusively identified using published literature.^11,19^ The *rpoB*-associated mutations were converted to the widely used *Escherichia coli* nomenclature (addition of 81 codon positions).^20^

### Resolving discordant phenotypic and WGS results

Discordant results were resolved using the minimum inhibitory concentration broth microdilution method (TREK Sensititre, Thermofisher, Waltham, Massachusetts, United States) and interpreted using the critical concentrations established by Hall et al. (2012).^21^ In the case of pyrazinamide, the modified Wayne’s test^22^ was used to resolve discordance. Additionally, the GenoType MTBDR*plus* assay version 2 (MTBDR*plus*) (Hain LifeSciences, Nehren, Germany) line probe assay was performed according to the manufacturer’s instruction for the first-line drugs rifampicin and isoniazid on all isolates. Figure 1 provides an overview of the operational workflow for this study.

## Results

Concordance between WGS and the phenotypic DST method for resistance determination was noted for all isolates except one phenotypically susceptible isolate for all targets explored (Table 1 and Table 2). The phenotypically susceptible isolate harboured a known resistance associated mutation in the fabG1/mabA (*inhA* promoter) region (*inhA* promoter ^c-15t^) detected by WGS. This finding was confirmed by the MTBDR*plus* assay displaying an *inhA*^mut1^ mutation, and resistance was confirmed by the broth microdilution assay (minimum inhibitory concentration of 0.25 *µ*g/ml) (Table 2). Interestingly, we found that a multidrug resistant isolate was incorrectly classified as susceptible to rifampicin by the MTBDR*plus* assay and resistant by both MGIT DST and WGS; the latter detected the presence of the *rpoB*^L511P^ mutation, a known rifampicin resistance determinant.

Three phenotypic discordant pyrazinamide isolates included were susceptible by WGS and susceptibility was confirmed by the modified Wayne’s test. Of note was the finding that one isolate had a *pncA*^Thr114Met^ mutation by WGS that was not listed in the TBDReaMDB database, and literature confirmed this not to be associated with resistance.^24^ Resolution testing using the modified Wayne’s test confirmed susceptibility.

The phenotypically discordant fluoroquinolone isolates (*n* = 3) were predicted to be susceptible by WGS, displaying a wild-type *gyrA* and *gyrB* gene. Repeat DST was in agreement with WGS for moxifloxacin; however, two of the three isolates remained resistant to ofloxacin. Resolution testing using the broth microdilution assay confirmed susceptibility for both isolates, showing a minimum inhibitory concentration of 1 *µ*g/ml.

WGS for novel drugs bedaquiline and delamanid showed no resistance-associated mutations.

## Discussion

The application of whole genome next-generation sequencing technology for drug resistance determination in *M. tuberculosis* has been shown to be a valuable tool in this study. Despite the small sample size, the performance of WGS for predicting resistance was consistent with published studies containing subsets of South African isolates.^25,26^

The use of the MiSeq^®^ offers reduced hands-on preparation time (~6 h per batch of isolates) compared to other next-generation sequencing technologies.^27^ Bioinformatic analysis using the commercial software was relatively straightforward, particularly the use of workflows for automation. Once a workflow is saved, imported data are automatically analysed, producing a final output table displaying mutations. However, sequence analysis requires an understanding of the associated genetic targets and drug resistance mutations.

Concordance of WGS with initial MGIT DST was lacking for isoniazid, pyrazinamide and fluoroquinolones; however, resolution testing improved agreement between WGS and phenotypic drug susceptibility profiles for the discordant isolates, even at a coverage of 5× (paired) with acceptable quality scores.

The use of this technology for resistance determination in *M. tuberculosis* is currently limited due to the lack of a comprehensive tuberculosis mutation catalogue predicting susceptibility, as seen in the case of the *pncA*^Thr114Met^ mutation in this study, which was not associated with resistance. Furthermore, sequence data can only be generated from cultured isolates, creating a lag between specimen receipt and a positive culture. Despite these limitations, WGS could benefit the majority of patients by enabling them to be placed on optimum regimens sooner in comparison to phenotypic methods.

### Limitations

The small sample size was inadequate for assessing diagnostic performance statistically. In addition, the data are based on a first attempt without any optimisation for sequence output.

### Conclusion

WGS correctly predicted resistance or susceptibility using commercial bioinformatics software based on already identified resistance-determining mutations. Our findings suggest the system shows promise as a tool for predicting drug resistance in a short time frame for multiple drugs and multiple samples simultaneously, provided the genetic basis for resistance is well described.
